# COMMUNITY DISADVANTAGE AND WORKFORCE PARTICIPATION AMONG LATE MIDDLE-AGED MEDICAID BENEFICIARIES

**DOI:** 10.1093/geroni/igac059.880

**Published:** 2022-12-20

**Authors:** Rodlescia Sneed

**Affiliations:** Wayne State University, Detroit, Michigan, United States

## Abstract

There have been numerous efforts to increase workforce participation among Medicaid beneficiaries in an effort to reduce program costs. We know little, however, about whether Medicaid beneficiaries with low workforce participation live in communities that support employment. The purpose of the current study was to determine if community disadvantage was associated with workforce participation among late middle-aged Medicaid beneficiaries, an age group that commonly faces difficulty entering the workforce. Participants were 1418 Medicaid beneficiaries ages 51-64 who participated in the 2016 wave of the Health and Retirement Study, a population-based study of community-dwelling adults aged >50. Community disadvantage was measured using the Social Vulnerability Index (SVI). We evaluated the association between community social vulnerability and reduced workforce participation (defined as working < 20 hours per week). We performed additional analyses to determine if the association between community disadvantage and reduced workforce participation was driven by any one of four subthemes of the SVI:(1) community socioeconomic status, (2) household composition/disability, (3) minority status & language and (4) housing type & transportation. Community disadvantages was associated with reduced workforce participation (OR: 2.82; 95% CI: 1.05-7.56). Analyses by SVI theme showed that the association between community disadvantage and reduced workforce participation was largely driven by community socioeconomic status (SES). Those living in low SES communities were more likely to report reduced workforce participation than those living in high SES communities: (OR: 5.48; 95% CI: 3.28-8.18). Policy interventions that focus on disadvantaged communities are likely needed to improve employment rates among late middle-aged Medicaid beneficiaries.

